# A Technology Acceptance Model for Deploying Masks to Combat the COVID-19 Pandemic in Taiwan (My Health Bank): Web-Based Cross-sectional Survey Study

**DOI:** 10.2196/27069

**Published:** 2021-04-21

**Authors:** Wen-Hsun Tsai, Yi-Syuan Wu, Chia-Shiang Cheng, Ming-Hao Kuo, Yu-Tien Chang, Fu-Kang Hu, Chien-An Sun, Chi-Wen Chang, Ta-Chien Chan, Chao-Wen Chen, Chia-Cheng Lee, Chi-Ming Chu

**Affiliations:** 1 Graduate Institute of Medical Sciences National Defense Medical Center Taipei Taiwan; 2 Medical Administration Office Beitou Branch Tri-Service General Hospital Taipei Taiwan; 3 Graduate Institute of Life Sciences National Defense Medical Center Taipei Taiwan; 4 School of Public Health National Defense Medical Center Taipei Taiwan; 5 Medical Informatics Office Tri-Service General Hospital Taipei Taiwan; 6 Big Data Research Center College of Medicine Fu-Jen Catholic University New Taipei City Taiwan; 7 School of Nursing College of Medicine Chang-Gung University Taoyuan Taiwan; 8 Division of Pediatric Endocrinology & Genetics Department of Pediatrics Chang-Gung Memorial Hospital Taoyuan Taiwan; 9 Research Center for Humanities and Social Sciences Academia Sinica Taipei Taiwan; 10 Trauma and Critical Care Service Department of Surgery Kaohsiung Medical University Hospital Kaohsiung Taiwan; 11 Division of Colorectal Surgery Department of Surgery Tri-Service General Hospital Taipei Taiwan; 12 Department of Public Health China Medical University Taichung Taiwan; 13 Department of Public Health College of Health Sciences Kaohsiung Medical University Kaohsiung Taiwan; 14 Department of Healthcare Administration and Medical Informatics College of Health Sciences Kaohsiung Medical University Kaohsiung Taiwan; 15 Department of Medical Research Kaohsiung Medical University Hospital Kaohsiung Taiwan

**Keywords:** personal health record, electronic medical record, my health bank, technology acceptance model, structural equation model, electronic health record, COVID-19, protection, survey, model, intention, usage, literacy, privacy, security

## Abstract

**Background:**

The successful completion of medical practices often relies on information collection and analysis. Government agencies and medical institutions have encouraged people to use medical information technology (MIT) to manage their conditions and promote personal health. In 2014, Taiwan established the first electronic personal health record (PHR) platform, My Health Bank (MHB), which allows people to access and manage their PHRs at any time. In the face of the COVID-19 pandemic in 2020, Taiwan has used MIT to effectively prevent the spread of COVID-19 and undertaken various prevention measures before the onset of the outbreak. Using MHB to purchase masks in an efficient and orderly way and thoroughly implementing personal protection efforts is highly important to contain disease spread.

**Objective:**

This study aims to understand people’s intention to use the electronic PHR platform MHB and to investigate the factors affecting their intention to use this platform.

**Methods:**

From March 31 to April 9, 2014, in a promotion via email and Facebook, participants were asked to fill out a structured questionnaire after watching an introductory video about MHB on YouTube. The questionnaire included seven dimensions: perceived usefulness, perceived ease of use, health literacy, privacy and security, computer self-efficacy, attitude toward use, and behavioral intention to use. Each question was measured on a 5-point Likert scale ranging from “strongly disagree” (1 point) to “strongly agree” (5 points). Descriptive statistics and structural equation analysis were performed using SPSS 21 and AMOS 21 software.

**Results:**

A total of 350 valid questionnaire responses were collected (female: 219/350, 62.6%; age: 21-30 years: 238/350, 68.0%; university-level education: 228/350, 65.1%; occupation as student: 195/350, 56.6%; average monthly income <NT $30,000 [<US $1054.89]: 230/350, 65.7%; residence in northern Taiwan: 236/350, 67.4%; and health status perceived as “good”: 171/350, 48.9%). Five indicators, including chi-square test (*X^2^*_310_=2.63), goodness-of-fit index (0.85), adjusted goodness-of-fit index (0.81), comparative fit index (0.91), and root mean square error of approximation (0.07), were calculated. The results indicated a good fit. Further analysis indicated that the most important factor affecting respondents’ behavioral intention to use MHB was their attitude toward use (0.78), followed by perceived ease of use (0.65), perceived usefulness (0.41), health literacy (0.10), and privacy and security (0.07).

**Conclusions:**

From the perspective of the populace, this study explored the factors affecting the use of MHB and constructed an interpretation model with a strong goodness of fit. The results of our analysis are consistent with the technology acceptance model. Through the diverse value-added services of MHB, Taiwan's experience in pandemic prevention with smart technology can facilitate future responses to unknown, emerging infectious diseases.

## Introduction

### Background

With the rapid development of various information and communication technologies (ICTs), medical institutions now use a variety of digital medical information tools to provide the information needed by patients and medical care providers and allow physicians and patients more time for communication and discussion to make correct medical decisions. Information tools are now regarded as the mainstream way to provide clinical care [1-3]. The digitization of personal health records (PHRs) is a particularly important tool for realizing the goal of patient-centered care [3-9]. PHRs can be collected in different forms for various health management purposes [10]. Compared with electronic medical records (EMRs), PHRs contain other health-related information in addition to medical records, such as social status, family history, and living environment [7,11-15]. Therefore, PHRs were built on the basis of EMRs. As the use of EMRs increases, the use of PHRs will also increase [11].

EMRs are mainly stored in hospital databases and can be accessed by different types of medical personnel but are not easily exchanged between hospitals. In 2004, Taiwan began to develop the basic format of EMRs to promote the digitization of health information. In 2007, Executive Yuan initiated the National Health Informatics Project to promote the development of a health care information infrastructure and create a development environment for health information [16]. In 2008, the Clinical Document Architecture was defined according to the Health Level 7 standard, and the localized EMR format—the Taiwan Electronic Medical Record Template—was created using 108 individual EMR templates, which provided an important foundation for the interhospital exchange of EMRs [7,17,18]. The Taiwanese governments have encouraged medical institutions to use EMRs through policies, regulations, and financial incentives for health care insurance.

Most studies on EMRs have been from the perspective of medical personnel. In a patient-centered paradigm, medical records would belong to the individual patient. Through EMRs, patients can have more control over their medical information. Zarcadoolas et al [5] used a focus group interview to explore the use of patient portals, which are health care–related web applications. Their results showed that most respondents were interested in accessing their own medical records and believed that such access was important in improving health literacy and promoting their own health and that of their family members. In a national study in the Netherlands, representatives of medical centers jointly discussed future medical information policies, among which “patient participation and empowerment” was given the highest score [19]. Therefore, for governments, the patients' acceptance of and intention to use electronic PHRs could influence subsequent policy formulation and must be considered. Owing to the advantages of EMRs, such as reducing medical costs, promoting care quality, and enhancing medical efficacy, many countries have begun to plan and develop EMRs with different formats and applications [20-24]. However, in most cases, patients cannot access and manage their PHR at any time. In view of this, the Ministry of Health and Welfare of Taiwan developed a web-based health information enquiry system, My Health Bank (MHB), in 2014 to allow people to access their personal health information from a computer or smartphone at any time or place [7,24-26].

The first edition of MHB could be used to check outpatient records, emergency records, inpatient records, dental records, traditional Chinese medicine records, disease diagnosis, drug usage, medical expenses, pathological reports, x-ray examinations, allergy histories, and vaccinations, but the interactivity and advanced query capabilities were limited. It also had no images. In other words, it only provided data, not information or knowledge [7,25,26]. Patient portals that present content in simple and easy-to-understand text or images are more in line with people’s internet usage habits; in addition, a user-friendly interface would help improve the utilization rate [5,26-28].

**Figure 1:**
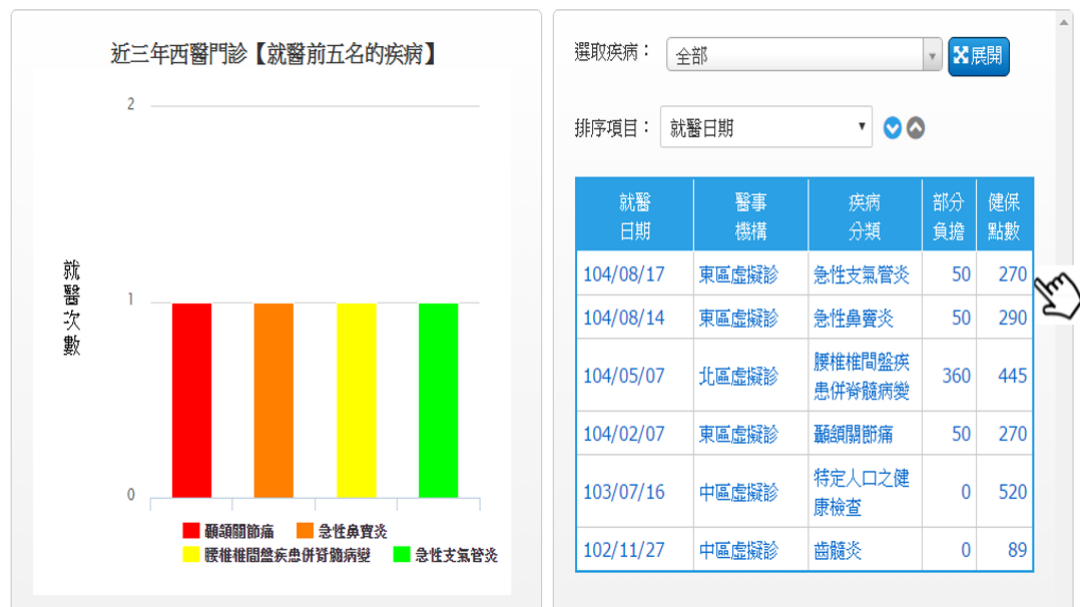
Medical data visualization (screenshot from My Health Bank): People can query information about outpatients (emergency department and hospital) and medical diagnoses over the past 3 years. Statistical results are presented graphically.

**Figure 2:**
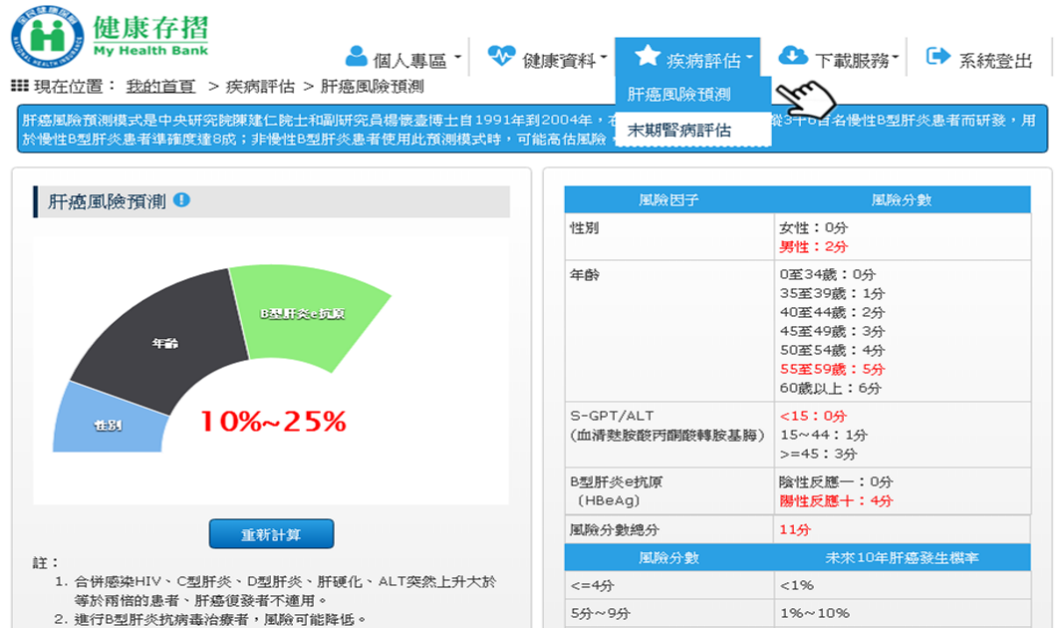
Disease management services (screenshot from My Health Bank): After the user enters their basic information, the system predict the risk of major diseases (eg, hepatic cancer and end-stage renal disease) and then provide links to external websites for further health information queries.

**Figure 3:**
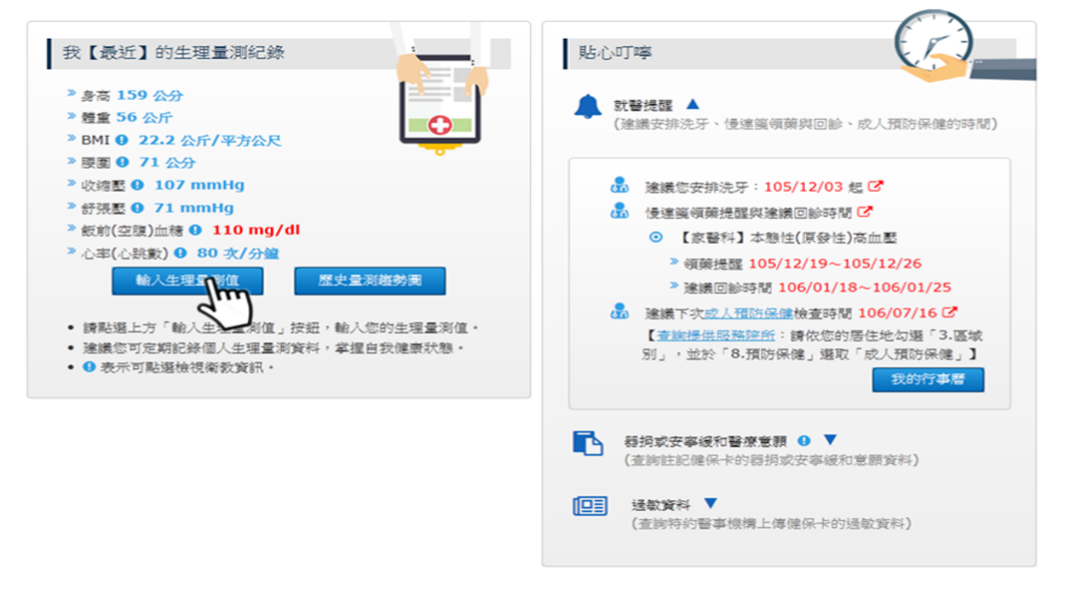
Self-health management (screenshot from My Health Bank): Physiological measurement data, such as height, weight, and blood pressure, are entered and historical trends are monitored. In addition, the system proactively reminds the user about dental cleaning, continuous prescription collection, and health check-ups.

**Figure 4:**
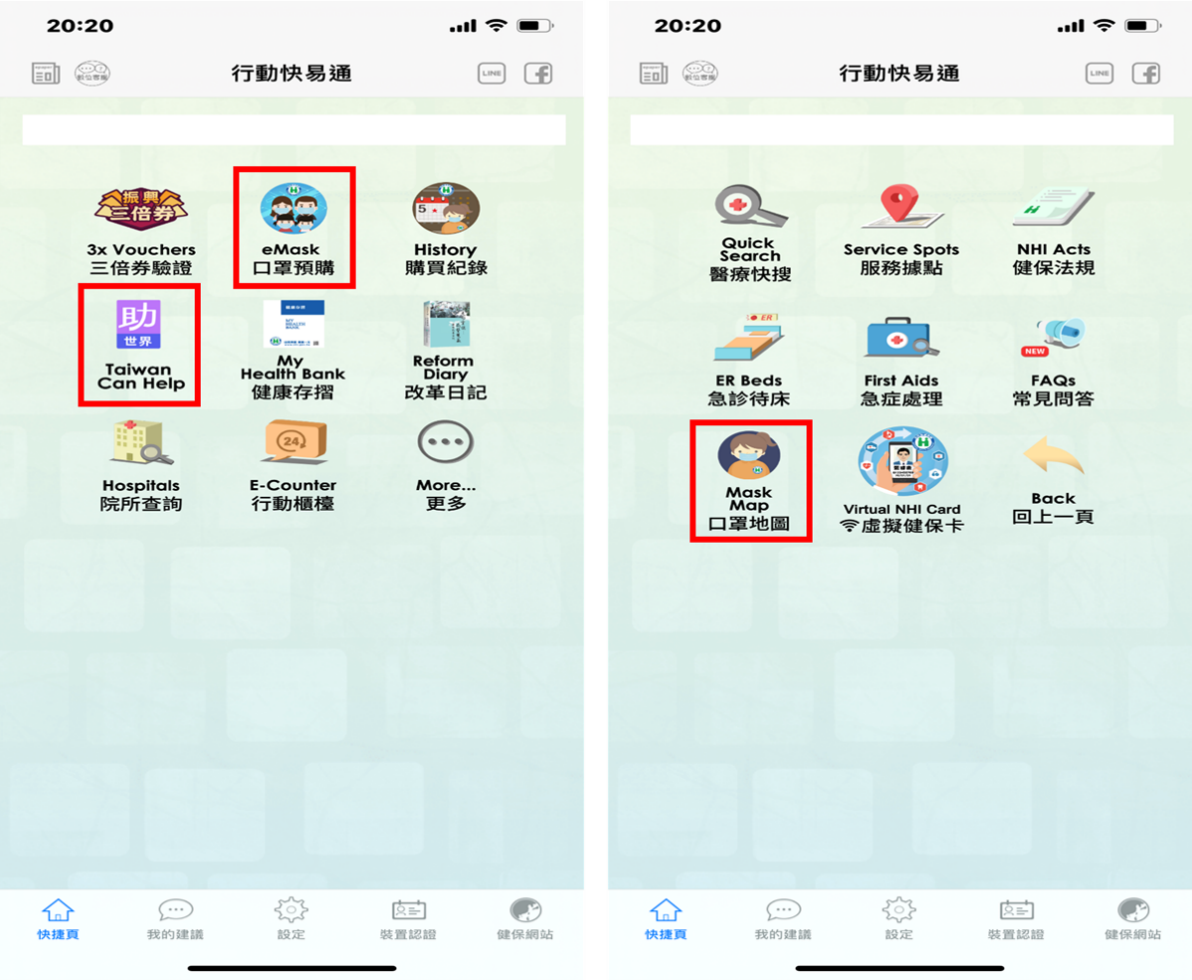
Preordering masks (screenshot from My Health Bank mobile app): Users can log in with their ID or mobile phone number to buy masks, check the stock of masks in nearby drugstores, or even donate their mask quotas to other countries.

MHB was thus revised in July 2016 with the addition of functions for visualization of medical information, disease management service, and self-health management, as shown in Figures 1-3 [26]. These new functions greatly improved the functionality of MHB and provided diversified health management services. In response to the need for functions specific to COVID-19 prevention, new functions such as preordering masks, maps of masks, and assistance with getting masks were added to MHB in March 2020, as shown in Figure 4 [29]. When the COVID-19 pandemic became severe, people in Taiwan could purchase masks through the MHB app, which not only helped them effectively implement personal protective measures but also helped stabilize public sentiments.

### Theoretical Foundation

With the development of informatization of the medical industry, the acceptance and use of relevant information technology by clinical medical staff and patients have gradually received attention. The research model used by most researchers is the technology acceptance model (TAM). Although it was not initially developed for use in the medical industry [30,31], this model has become an important theoretical model for studying medical information usage behavior with the expansion of research on the medical industry in recent years [32-40]. In the original TAM, Davis et al [30,31] used three variables: “perceived usefulness,” “perceived ease of use,” and “attitude toward use” to explain and predict the behavioral intention of users. Because health behaviors are too complex to be explained by a single theory, many researchers use TAM as a foundation in combination with other theories or references to construct theoretical models with better explanatory capabilities in the form of added variables [31,33,36,37,40-46].

According to a literature review by Rahimi et al [45], the original TAM has been extended to suit the dynamic health service environment. TAM has been used to explore three categories of application areas of ICT in the medical service industry: “telemedicine,” “electronic health records,” and “mobile applications.” Researchers have added different variables to the original TAM model according to different ICT application fields, thereby enhancing the explanatory power of the extended model. Health literacy is a predictive factor that has been widely studied in research on health-related behaviors and is also a key factor for the appropriate selection and use of health information [42,47]. Relevant studies have also found that the application of health literacy in TAM is related to perceived usefulness, perceived ease of use, and behavioral intention. A high level of health literacy can increase people’s willingness to adopt new health information technology [12,42,48,49].

The health system is a complex social system composed of stakeholders with different backgrounds, experiences, and values [36]. ICT applications allow the system to run smoothly. The storage, retrieval, transmission, and sharing of medical information are closely related to the operation of computer software and hardware [31]. Compeau and Higgins defined “computer self-efficacy” as an individual's ability to use information technology, which plays an important role in shaping personal perception and usage behavior. Computer self-efficacy also affects an individual’s perceived ease of use. Individuals with high computer self-efficacy use computers more often and have less computer anxiety [44,46,50-52]. However, in the process of using medical information technology (MIT), new issues of security and privacy may arise [41]. To solve these security and privacy issues, many countries have not only enacted laws and regulations to protect their citizens’ health data [53] but also studied and explored the impact of security and privacy on the use of mobile health (mHealth) systems [41,45,54,55].

This study explores the intention of the Taiwanese people to use MHB and the factors influencing this intention, thus providing a reference for governments to consult when promoting electronic PHRs in the future.

## Methods

### Study Design

**Figure 5:**
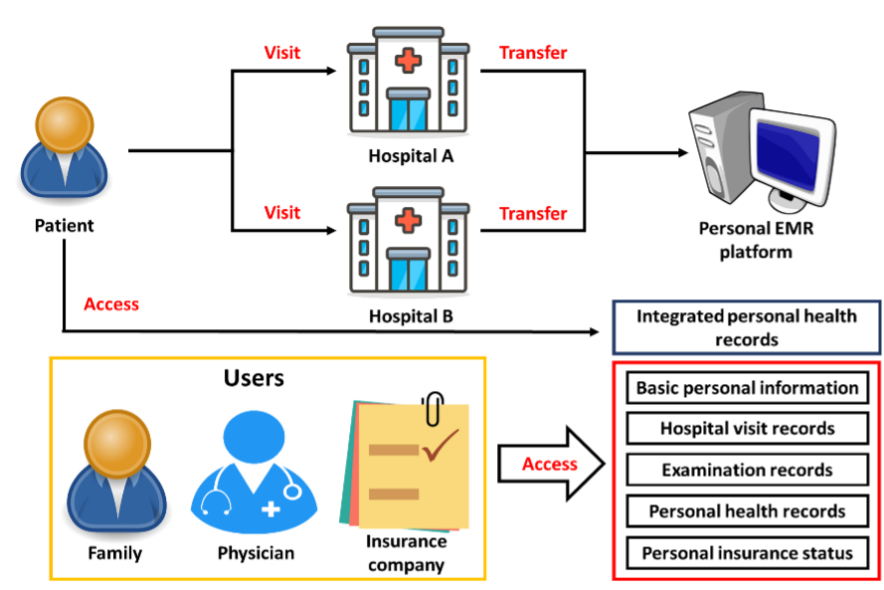
Schematic of My Health Bank.

This was a cross-sectional study. To investigate participants’ intention to use MHB and the influencing factors, participants were asked to watch a brief introductory video about MHB on YouTube and then fill out a structured questionnaire on Google Forms. The content of the video was how to use MHB, as shown in Figure 5. After a patient goes to hospital A for medical treatment, he or she can log into MHB with their account number, password, or natural person certificate to access five items: basic personal information, hospital visit records, examination records, PHRs, and personal insurance status. This information can be shared with other medical personnel, family members, and insurance companies.

**Figure 6:**
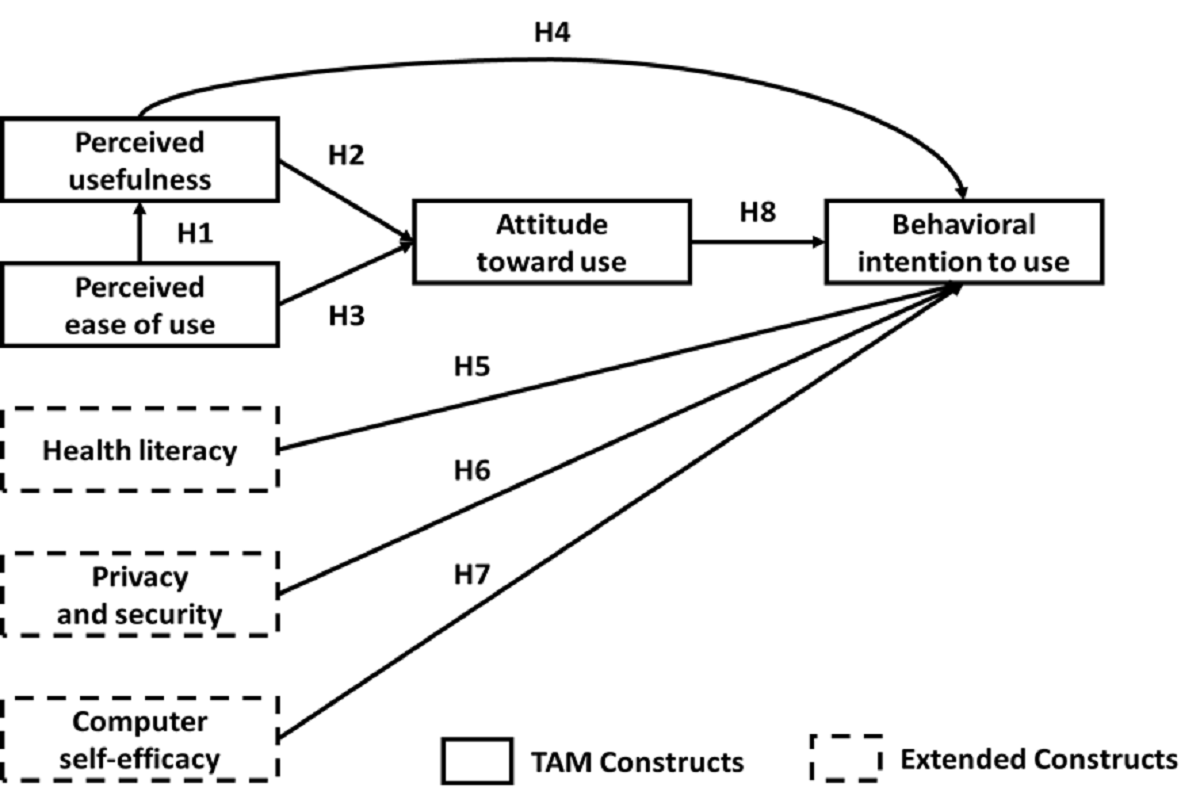
Research framework and hypotheses (extension of the technology acceptance model). EMR: electronic medical record.

Based on the above study objectives and a literature review, we added three extended variables to the original technology acceptance model: “Health literacy,” “Privacy and security,” and “Computer self-efficacy.” The research framework and hypotheses are illustrated in Figure 6 and described below:

- Hypothesis 1: Perceived ease of use has a positive effect on perceived usefulness.
- Hypothesis 2: Perceived usefulness has a positive effect on attitude toward use.
- Hypothesis 3: Perceived ease of use has a positive effect on attitude toward use.
- Hypothesis 4: Perceived usefulness has a positive effect on behavioral intention to use.
- Hypothesis 5: Health literacy has a positive effect on behavioral intention to use.
- Hypothesis 6: Privacy and security have a positive effect on behavioral intention to use.
- Hypothesis 7: Computer self-efficacy has a positive effect on behavioral intention to use.
- Hypothesis 8: Attitude toward use has a positive effect on behavioral intention to use.

### Research Tools

Table 1 shows the questionnaire comprising 33 questions that covered seven dimensions: perceived usefulness, perceived ease of use, health literacy, privacy and security, computer self-efficacy, attitude toward use, and behavioral intention to use. Each question was answered on a 5-point Likert scale (“Strongly agree,” 5 points; “Agree,” 4 points; “Neutral,” 3 points; “Disagree,” 2 points; and “Strongly disagree,” 1 point).

Before the formal survey, four experts in relevant fields were invited to assess the importance, applicability, and clarity of each question in the questionnaire to confirm that the questionnaire had good external validity. In addition, a pretest was conducted with 30 people to confirm that respondents could understand the questions and answer them clearly.

**Table 1:** Questionnaire design.

Dimension	Definition	Questions
Perceived usefulness	The usefulness of MHB^a^ in managing the PHR^b^	5
Perceived ease of use	The ease of use of MHB in managing the PHR	5
Health literacy	When using MHB, the required ability to process and understand this information service in order to make appropriate medical and health decisions	3
Privacy and security	The awareness of privacy and security issues when using MHB	5
Computer self-efficacy	The basic computer skills required when using MHB	5
Attitude toward use	Public evaluation of MHB	4
Behavioral intention to use	The intention to use MHB	6

^a^MHB: My Health Bank.

^b^PHR: personal health record.

### Sampling and Exclusion Criteria

From March 31 to April 9, 2014, in a promotion via email and Facebook, internet users were randomly selected to watch the introductory video about MHB and then complete the web-based questionnaire. The size of the valid sample was determined according to the sampling guidelines proposed by Magnani et al [56,57]. The calculated minimum number of valid samples needed for this study was 323. During the collection process, 614 people visited the website, 355 of whom completed the questionnaire (response rate 57.8%). After excluding duplicate and invalid questionnaire responses, 350 questionnaire responses were collected, which exceeded the minimum number of valid samples needed for this study.

### Data Analysis

The data in this study were sorted in Microsoft Excel and then analyzed with SPSS 21 (IBM Corp). The significance level for statistical analysis was set at 5%, that is, *P*<.05. Descriptive statistics, including the mean, SD, median, frequency distribution, and percentage, were used to observe the data distribution of the responses.

Inferential statistics were used to understand the correlations between the characteristics of the respondents and perceived usefulness, perceived ease of use, health literacy, privacy and security, computer self-efficacy, attitude toward use, and behavioral intention to use. AMOS 21 (IBM Corp) was used to test the validity of the dimensions and construct the research model.

## Results

### Respondent Characteristics

A total of 350 valid questionnaires were collected in this study, and the distribution of the respondents’ characteristics is shown in Table 2. Most respondents were female (219/350, 62.6%). The age grouping interval was 10 years, and the majority of the respondents (238/350, 68%) were in the age group of 21-30 years. The majority of the respondents had a university education (228/350, 65.1%), were students (195/350, 55.7%), had an average monthly income lower than NT $30,000 (US $1054.89; 230/350, 65.7%), resided in northern Taiwan (236/350, 67.4%), and had a self-perception of *good* health (171/350, 48.9%).

**Table 2:** Characteristics of the respondents (N=350).

Characteristic			Value, n (%)
**Sex**			
	Male	131 (37.4)	
	Female	219 (62.6)	
**Age (years)**			
	<20	46 (13.1)	
	21-30	238 (68)	
	31-40	56 (16.)	
	>40	10 (2.9)	
**Highest education level**			
	High school degree	8 (2.3)	
	University degree	228 (65.1)	
	Graduate school or above	114 (32.6)	
**Employment**			
	Student	195 (55.7)	
	Services	61 (17.4)	
	Manufacturing	29 (8.3)	
	Financial industry	7 (2)	
	Military and police education	47 (13.4)	
	Unemployed	11(3.1)	
**Monthly income in NT $ (US $)**			
	<30,000 (<1054.89)	230 (65.7)	
	30,001-50,000 (1054.92-1758.15)	94 (26.9)	
	50,001-70,000 (1758.18-2461.41)	21 (6)	
	>70, 001 (>2461.44)	5 (1.5)	
**Living area**			
	Northern Taiwan	236 (67.4)	
	Central Taiwan	49 (14)	
	Southern Taiwan	55 (15.7)	
	Eastern Taiwan	7 (2)	
	Offshore islands	3 (0.9)	
**Health status**			
	Excellent	28 (8)	
	Good	171 (48.9)	
	Normal	132 (37.7)	
	Poor	19 (5.4)	

### Measurement Model

This study evaluated the measurement model with internal reliability, convergent validity, and discriminant validity [58]. Internal reliability was evaluated by Cronbach alpha and composite reliability. A value higher than .70 can be regarded as an acceptable level of internal consistency [59]. If the “average variance extracted” (AVE) for a dimension is higher than 0.50, the model is deemed to have reached acceptable convergent validity. Table 3 shows the composite reliability, Cronbach *α*, and AVE obtained in this study.

Discriminant validity was evaluated by calculating the square root of AVE and cross-loading matrix. When the square root of the AVE is greater than the corresponding correlation, the discriminant validity of the data is confirmed. The calculated values are shown in Table 4.

**Table 3:** Cronbach alpha, composite reliability, and average variance extracted.

Dimension	Cronbach *α*	Composite reliability	Average variance extracted
PU^a^	.85	0.82	0.53
PE^b^	.83	0.81	0.52
HL^c^	.82	0.70	0.54
PS^d^	.95	0.90	0.75
CE^e^	.78	0.80	0.50
AU^f^	.86	0.84	0.57
BI^g^	.92	0.93	0.69

^a^PU: perceived usefulness.

^b^PE: perceived ease of use.

^c^HL: health literacy.

^d^PS: privacy and security.

^e^CE: computer self-efficacy.

^f^AU: attitude toward use.

^g^BI: behavioral intention to use.

**Table 4:** Correlation matrix and square root of the average variance extracted. Note: The elements on the diagonal represent the square root of the average variance extracted, and off-diagonal elements represent the correlations between the constructs.

Dimension	PU^a^	PE^b^	HL^c^	PS^d^	CE^e^	AU^f^	BI^g^
PU	0.73	0.72	0.24	0.10	0.59	0.71	0.62
PE	0.72	0.72	0.45	0.24	0.69	0.71	0.72
HL	0.24	0.45	0.74	0.36	0.48	0.26	0.33
PS	0.10	0.24	0.36	0.87	0.33	0.30	0.19
CE	0.59	0.69	0.48	0.33	0.71	0.70	0.65
AU	0.71	0.71	0.26	0.30	0.70	0.75	0.74
BI	0.62	0.72	0.33	0.19	0.65	0.74	0.83

^a^PU: perceived usefulness.

^b^PE: perceived ease of use.

^c^HL: health literacy.

^d^PS: privacy and security.

^e^CE: computer self-efficacy.

^f^AU: attitude toward use.

^g^BI: behavioral intention to use.

### Structural Model

Structural equation modeling is a multivariate statistical technique combining factor analysis and path analysis [60]. In this study, we used structural equation modeling to investigate the relevant factors affecting participants’ use of MHB and the importance of each factor [34]. Five indicators were used to assess the overall fit of the model: *Χ^2^* (*df*), goodness-of-fit index (GFI), adjusted goodness-of-fit index (AGFI), comparative fit index (CFI) and root mean square error of approximation (RMSEA). As shown in Table 5, *Χ^2^*_310_=2.63, which is less than the cutoff of 5 [61]; GFI=0.85 and AGFI=0.81, both of which are higher than the standard value of 0.8 [62,63]; CFI=0.91, which is close to 1 [64]; and RMSEA=0.07 [65]. The above results indicate that the overall fit and fitness of the model are good.

**Table 5:** Results of overall model fit.

Index	Criteria	Result	Mode fit
Chi-square (*df*)	<5	2.63 (310)	Fit
Goodness-of-fit index	>0.8	0.85	Fit
Adjusted goodness-of-fit index	>0.8	0.81	Fit
Comparative fit index	Close to 1	0.91	Fit
Root mean square error of approximation	Acceptable: 0.08-0.10 Good: 0.05-0.08 Excellent: <0.05	0.07	Good

**Figure 7:**
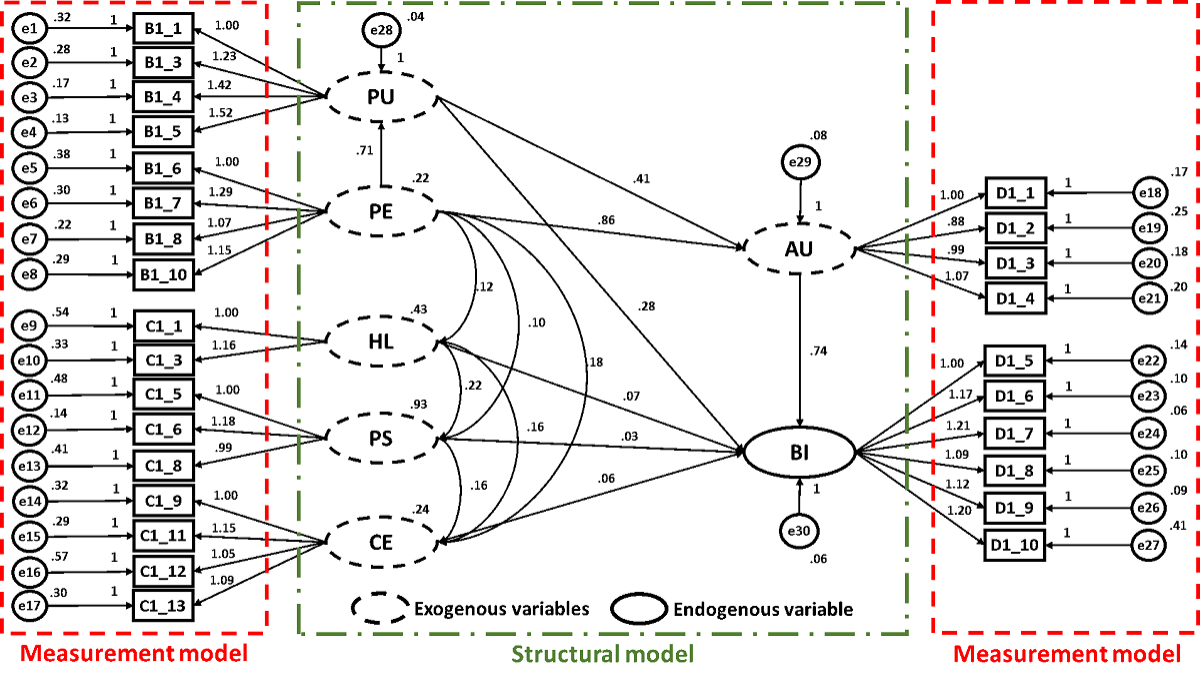
Path diagram of the research framework. Measured items are illustrated in rectangles (eg, B1_1); latent variables are illustrated in ovals (eg, PU); smaller circles illustrate the error of measurement (eg, e1); associations are illustrated by arrows that indicate the direction of prediction. Factor loadings are noted next to the items. Coefficients are noted for each association (ie, directional arrow). AU: attitude toward use; BI: behavioral intention to use; CE: computer self-efficacy; HL: health literacy; PE: perceived ease of use; PS: privacy and security; PU: perceived usefulness.

Structural equation modeling was used to evaluate whether the causal path relationships between the hypotheses proposed in this study were valid. The path diagram is shown in Figure 7, and the standardized estimation results are shown in Table 6. Perceived ease of use explained 74.7% of the variance in perceived usefulness. Perceived usefulness and perceived ease of use explained 67.4% of the variance in attitude toward use, and the other dimensions explained 72.6% of the variance in behavioral intention to use. The above results are the direct effects between the dimensions. To further understand the effects between dimensions other than direct effects, indirect effects were included in the analyses to calculate the total effects. Table 7 shows the standardized direct effects, indirect effects, and total effects of the dimensions.

In terms of behavioral intention to use, the direct effect of attitude toward use (0.78) was relatively large, whereas perceived ease of use (0.65) and perceived usefulness (0.13) had significant indirect effects on behavioral intention to use. Perceived ease of use had no direct effect on behavioral intention to use, and its indirect effect was much stronger than the direct effects of the other dimensions. Overall, the main factor affecting behavioral intention to use was the attitude toward use, followed by perceived ease of use, perceived usefulness, health literacy, and privacy and security. The test results of all hypotheses were statistically significant except for Hypothesis 5, Hypothesis 6, and Hypothesis 7.

**Table 6:** Results of path analysis.

Hypothesis	Path coefficient	*t* test (*df*)	*P* value	*R^2^*
H1: Perceived ease of use has a positive effect on perceived usefulness.	0.71	8.24 (310)	<.001	0.747
H2: Perceived usefulness has a positive effect on attitude toward use.	0.41	3.12 (310)	<.001	0.674
H3: Perceived ease of use has a positive effect on attitude toward use.	0.86	5.01 (310)	<.001	—^a^
H4: Perceived usefulness has a positive effect on behavioral intention to use.	0.28	3.49 (310)	<.001	0.726
H5: Health literacy has a positive effect on behavioral intention to use.	0.07	1.83 (310)	.067	—
H6: Privacy and security has a positive effect on behavioral intention to use.	0.03	1.58 (310)	.115	—
H7: Computer self-efficacy has a positive effect on behavioral intention to use.	0.06	0.73 (310)	.468	—
H8: Attitude toward use has a positive effect on behavioral intention to use.	0.74	8.18 (310)	<.001	—

^a^Not available.

**Table 7:** Direct effects, indirect effects, and total effects.

Dimension and effect		PU^a^	PE^b^	HL^c^	PS^d^	CE^e^	AU^f^	BI^g^
**PU**								
	Direct effect	—^h^	0.86	—	—	—	—	—
	Indirect effect	—	—	—	—	—	—	—
	Total effect	—	0.86	—	—	—	—	—
**AU**								
	Direct effect	0.28	0.81	—	—	—	—	—
	Indirect effect	—	0.02	—	—	—	—	—
	Total effect	0.28	0.82	—	—	—	—	—
**BI**								
	Direct effect	0.28	—	0.10	0.07	0.06	0.78	—
	Indirect effect	0.13	0.65	—	—	—	—	—
	Total effect	0.41	0.65	0.10	0.07	0.06	0.78	—

^a^PU: perceived usefulness.

^b^PE: perceived ease of use.

^c^HL: health literacy.

^d^PS: privacy and security.

^e^CE: computer self-efficacy.

^f^AU: attitude toward use.

^g^BI: behavioral intention to use.

^h^Not available.

## Discussion

### Principal Findings

Taiwan has had no local cases of COVID-19 since April 12, 2020, and there has been no second wave of the pandemic in Taiwan, which is mainly due to the effective use of MIT to build a strong pandemic prevention network. For example, an integrated circuit card is used as an insurance certificate to give full play to the functions of smart medical cards. Physicians can log into the health care medical information cloud query system through a health insurance card to quickly obtain the medical information, travel history, and contact history of patients without revealing personal information. During the COVID-19 pandemic, assisting frontline medical personnel in assessing disease risk and taking corresponding infection control measures has been greatly helpful for prevention of disease spread [66]. In addition, in accordance with the policy of real-name mask purchasing, people who have completed identity authentication or mobile phone authentication can use MHB to prepurchase masks and know how many masks are still available to them from the mask purchase map, so they can purchase personal protective equipment more efficiently. The aforementioned innovative information technologies, together with the National Health Insurance and the Central Epidemic Command Center, which conducts daily live broadcasts to explain the pandemic situation and future pandemic prevention policies, has calmed the unease and anxiety of the people and made pandemic control in Taiwan effective [67].

During the promotion of an innovative medical information service, users might experience the three stages of awareness, want, and adoption. Liang [2] found that there is a digital divide between these three stages. This digital divide differs significantly not only between demographic groups but also depending on personal computer ownership and internet use habits. Therefore, the acceptance of an innovative medical information service by users could play a key role in user attitude and experience [7].

This study found that perceived ease of use had a significant positive effect on perceived usefulness, indicating that the respondents believed that if MHB was easy to use, its effects could be significant. Perceived usefulness and perceived ease of use had significant positive impacts on the attitude toward use, indicating that the respondents believed that if MHB had practical benefits in managing personalized EMRs and was easy to use, they would have a positive attitude toward the use of MHB. Perceived usefulness had a significant positive impact on behavioral intention to use, which means that the respondents believed that if MHB helped them understand their own health conditions, their intention to use MHB would increase. Attitude toward use had a positive effect on behavioral intention to use, indicating that the better the functions of respondent’s health records are managed, the higher their intention to use MHB.

Financial and resource constraints on the health care system are increasing due to the aging population and changing disease patterns [68]. To improve the service efficiency of medical institutions and the participation of patients, medical institutions are encouraging patients to make appropriate medical care decisions and undertake health promotion activities through electronic PHRs to control their own health status and achieve the goal of patient-centered care [9,27,69]. Therefore, increasing patients' intention to use electronic PHRs is particularly important. The TAM proposed by Davis et al [30] indicated that perceived ease of use affects perceived usefulness and, thus, intention to use. The results of this study showed that respondents strongly agreed (5=“strongly agree,” 1=“strongly disagree) with the usefulness (mean score 4.42, SD 0.53) and ease of use of MHB (mean score 4.28, SD 0.57), and these two technical or cognitive factors had a significant positive effect on the respondents’ attitude toward use and behavioral intention to use concerning MHB. Other studies have shown that both the general public and clinicians are more willing to use a medical information tool if the interface of the tool is designed to be easy to use and can fully realize the benefits of care [18,27,70-72]. MHB has become an extension of the medical treatment process and an important management tool to improve health knowledge and promote the health of people and their loved ones [5].

According to a web-based survey on health issues, 70% of people and 65% of physicians believe that patients can download and manage their personalized digital health information [73]. When people use digital health information tools, in addition to a certain ability to use information devices, they must also be able to understand the health information itself. In theory, with advances in information technology, the effectiveness of various health care services may also increase, but people with low computer self-efficacy and health literacy may not be able to make full use of the information technology, which could further deepen the digital divide [1,4,5,19,28,74]. Therefore, to effectively use personalized digital health information, people should have sufficient health literacy and computer self-efficacy. The World Health Organization defines health literacy as “the achievement of a level of knowledge, personal skills and confidence to take action to improve personal and community health by changing personal lifestyles and living conditions” [75]. In summary, health literacy refers to how people acquire, understand, use, and communicate health-related information [42,49]. Computer self-efficacy is self-judgment and self-confidence in the ability to use a computer and includes both computer operation skills (such as formatting hard disks) and the ability to combine these skills to perform different tasks [52]. In this study, health literacy and computer self-efficacy did not have a significant positive impact on behavioral intention to use MHB, indicating that the respondents' intention to use MHB would not be affected by their own health literacy and computer self-efficacy. Due to advances in information technology and higher education levels, the health literacy and computer self-efficacy of young people are higher than those of older people [5,76-78]. The respondents in this study were mostly young internet users who likely had confidence in understanding, acquiring, and applying relevant health information. Therefore, their levels of health literacy and computer self-efficacy did not affect their intention to use MHB. Nevertheless, the impact of health literacy and computer self-efficacy on the use of medical information tools cannot be ignored.

The promotion and use of various MITs are aimed at improving the quality of care and patient safety, enabling correct medical decision-making, reducing medical costs, improving the accessibility of medical services, and promoting service efficiency [1,2,7-9,18,27,69,74,79-81]. In the context of using these MITs, privacy and security are relatively important issues. Studies have shown that regardless of country or national conditions, people’s views on the privacy and security of health information systems are consistent [23,24,73]. Since the discussion of medical privacy is a relatively abstract concept, this study evaluated privacy issues in PHRs from three aspects: information privacy, psychological privacy, and social privacy [82]. Privacy and security had no influence on the behavioral intention to use MHB. Due to the increasing popularity of social media (eg, Facebook, blogs, and Twitter), people care more about the speed of information transmission than about how to protect their privacy [83]; in addition, respondents who have not actually used MHB may have different views on privacy and security. Although our results on privacy and security did not reach statistical significance, the scores of the privacy and security dimension showed that most respondents chose the option “disagree” (mean score 2.45, SD 1.07), indicating that the respondents were still concerned about data privacy and security issues. In the future establishment and promotion of digital PHRs, privacy and security should be given continuous attention [4,6,23,84,85].

In addition, the results of this study show that attitude toward use was the main factor affecting behavioral intention to use (total effect: 0.78), and the Likert scores of the respondents on attitude toward use (mean 4.22, SD 0.56) and behavioral intention to use (mean 4.20, SD 0.56) were positive. The results of our path analysis showed that attitude toward use had a positive effect on behavioral intention to use. Therefore, our results confirm that when the attitude toward use is more positive, the intention to use is higher. To enhance the public's views on the ease of use and usefulness of MHB and to strengthen the positive assessment of MHB, the Ministry of Health and Welfare should continue to design diversified value-added services for MHB.

Since March 2020, with the policy of real-name mask purchases, MHB has combined the functions of mask preorders, mask purchase maps, and mask donation. The Taiwanese people can conveniently purchase masks during the COVID-19 pandemic to achieve effective COVID-19 prevention [29,86]. Combined with its excellent public health facilities, Taiwan took advantage of its advanced deployment of MIT to prevent and control the COVID-19 pandemic at the early stage. In contrast to the severe ongoing global COVID-19 pandemic, Taiwan has seen a respite from the impact of the disease [67,87]. In 2017, approximately 590,000 MHB accounts were registered and used, accounting for approximately 2.7% of the total Taiwanese population [24]. After adding a variety of diverse services in MHB, according to Apple, there were more than 7.3 million downloads of MHB in 2020, and the number of users significantly increased by 31.7% [88], which shows that as long as people are satisfied with the real benefits and value brought by an information tool, their intention to use it will increase, and the actual use of the tool will increase accordingly.

### Limitations

The MHB discussed in this study is a newly promoted health information service in Taiwan, and relatively few people have used it before. Therefore, people might have a limited understanding of the service, content, and functions of MHB. To reduce possible errors in our study, before filling out the questionnaire, the respondents were asked to watch an introductory video about MHB to ensure that they had a certain level of knowledge about the service.

In this study, a structured web-based questionnaire was used to survey the people, so the results may not cover the entire parent population, and the sample representativeness may be limited. Most of the respondents were young female internet users and lived in northern Taiwan. Therefore, future studies should collect more samples to increase the representativeness of the results. In addition, respondents may be affected by the surrounding environment, emotions, and other uncertain factors, resulting in measurement errors, which can be corrected using appropriate statistical methods.

In this study, data were collected by means of a cross-sectional survey, a method that is fast, easy, and inexpensive to perform and the results of which are helpful for further complex research [89]. A study that used longitudinal methods to assess the 14-year use of clinical information systems found that the acceptance of clinical information systems increased over time. In addition, the factors affecting acceptance varied with time [38]. Accordingly, with the development of the life cycle of ICT applications, the relationship between variables in TAM may also change [45]. Therefore, future studies may also consider regularly collecting data to investigate the effect of time factors on the use of MHB. Although it is difficult to extrapolate the results, this pioneering study could still be used as a reference for the development of electronic PHR platforms in Taiwan.

### Conclusions

This study used the TAM and a structured web-based questionnaire to investigate the Taiwanese people’s intention to use MHB. Validation by structural equation modeling was performed to obtain an interpretation model with good fitness, and the results showed that perceived ease of use has a significant positive impact on perceived usefulness; perceived ease of use and perceived usefulness both have a significant positive influence on attitude toward use; perceived usefulness has a significant positive impact on behavioral intention to use; and attitude toward use has a significant positive effect on behavioral intention to use. Health literacy, computer self-efficacy, and privacy and security have nonsignificant effects on behavioral intention to use. Further exploration of the effectiveness of each dimension indicates that attitude toward use is the most important dimension affecting respondents’ use of MHB. Even though health literacy, computer self-efficacy, and privacy and security have no significant impact on intention to use, they are still considered important influencing factors in relevant studies. In the future, different research designs can be used for further exploration.

The purpose of establishing MHB is to return the management of personal health information to patients so that they can check their past medical experience and health records at any time to strengthen their self-health care ability. In addition, MHB also allows patients to have autonomy over their personal digital health records, which truly realizes the idea of empowering the people. The planning of a nation's medical policy should be adjusted with the development of the latest ICTs and must be able to accommodate all kinds of complex scientific and humanity issues. Policy implementation not only enables disease management from the perspective of individuals but also promotes health value from the perspective of groups. Taiwan's successful experience in preventing the COVID-19 pandemic by using various MITs is conducive to the sustainable development of more diversified value-added services for MHB in the future and the creation of different medical service modes. The model developed in this study could not only be applied to the adoption of other similar PHR platforms but also be used as a reference for other countries to formulate medical information policies to provide management insights, thereby increasing the use of patients' PHRs.

## Abbreviations

AGFI: adjusted goodness-of-fit index
EMRs: electronic medical record
GFI: goodness-of-fit index
ICTs: information and communication technologies
MHB: My Health Bank
mHealth: mobile health
MIT: medical information technology
PHRs: personal health records
RMSEA: root mean square error of approximation
TAM: technology acceptance model
